# Learning inguinal hernia repair? A survey of current practice and of preferred methods of surgical residents

**DOI:** 10.1007/s10029-020-02270-y

**Published:** 2020-09-05

**Authors:** T. Nazari, M. E. W. Dankbaar, D. L. Sanders, M. C. J. Anderegg, T. Wiggers, M. P. Simons

**Affiliations:** 1 Department of Surgery, Erasmus University Medical Center, Rotterdam, The Netherlands; 2 The Institute of Medical Education Research Rotterdam (iMERR), Rotterdam, The Netherlands; 3 Department of Education, Erasmus University Medical Center, Rotterdam, The Netherlands; 4 North Devon District Hospital, Barnstaple, UK; 5 Department of Surgery, Amsterdam University Medical Centers, Amsterdam, The Netherlands; 6 Incision Academy, Amsterdam, The Netherlands; 7 Department of Surgery, OLVG, Amsterdam, The Netherlands

**Keywords:** Surgical education, Liechtenstein, Open inguinal hernia repair, Endoscopic inguinal hernia repair

## Abstract

**Purpose:**

During surgical residency, many learning methods are available to learn an inguinal hernia repair (IHR). This study aimed to investigate which learning methods are most commonly used and which are perceived as most important by surgical residents for open and endoscopic IHR.

**Methods:**

European general surgery residents were invited to participate in a 9-item web-based survey that inquired which of the learning methods were used (checking one or more of 13 options) and what their perceived importance was on a 5-point Likert scale (1 = completely not important to 5 = very important).

**Results:**

In total, 323 residents participated. The five most commonly used learning methods for open and endoscopic IHR were apprenticeship style learning in the operation room (OR) (98% and 96%, respectively), textbooks (67% and 49%, respectively), lectures (50% and 44%, respectively), video-demonstrations (53% and 66%, respectively) and journal articles (54% and 54%, respectively). The three most important learning methods for the open and endoscopic IHR were participation in the OR [5.00 (5.00–5.00) and 5.00 (5.00–5.00), respectively], video-demonstrations [4.00 (4.00–5.00) and 4.00 (4.00–5.00), respectively], and hands-on hernia courses [4.00 (4.00–5.00) and 4.00 (4.00–5.00), respectively].

**Conclusion:**

This study demonstrated a discrepancy between learning methods that are currently used by surgical residents to learn the open and endoscopic IHR and preferred learning methods. There is a need for more emphasis on practising before entering the OR. This would support surgical residents’ training by first observing, then practising and finally performing the surgery in the OR.

**Electronic supplementary material:**

The online version of this article (10.1007/s10029-020-02270-y) contains supplementary material, which is available to authorized users.

## Introduction

Inguinal hernia repair (IHR) is one of the first surgical procedures that surgical residents learn during their training [1], as it is a relatively simple surgical procedure to familiarise residents with the importance of understanding surgical anatomy and essential surgical skills. The European Hernia Society’s updated guideline for the treatment of inguinal hernia in adult patients recommends either the open or laparo-endoscopic approach—providing the surgeon has expertise in that approach—as best-evidence based options for IHR [2, 3]. The open IHR is easier to teach surgical residents compared to the endoscopic IHR [4] and fewer surgical procedures are required for proficiency [5].

The training of surgical residents is evolving from the traditional “see one, do one, teach one” model towards preparation before stepping into the operating room (OR) [6]. One of the reasons being the duty hour restriction which has led to less exposure time in the OR [7] and decreasing educational outcomes [8]. Additionally, patient safety and the general opinion not to practice on patients forces surgical training to change. Surgical residents can learn complex skills in a variety of ways. Knowledge can be learned using books, articles, lectures, videos or e-learnings [9]; skills can be trained in a simulation setting [10], followed by performing the surgical procedure in the OR, with repeated practice and feedback.

Basically, these learning methods aim to improve surgical performance to a level of proficiency. The surgical performance can be assessed by many available yet resource-intense tools. Therefore, the number of surgical procedures performed is commonly used as a proxy for proficiency [5]. Also, operative time [11] or complication rates [12] can be used. The extent of proficiency experienced by surgical residents reflects their confidence and knowledge level; however, to our knowledge, no information is available on when surgical residents consider themselves to be proficient for the IHR.

Even though the aforementioned stages to learn complex skills and achieve proficiency are known—observing, practising, performing and receiving feedback—it is unclear which learning methods—aiming both at theoretical learning and skills learning—are in fact most commonly used by residents, and which are perceived as most important for open and endoscopic IHR. This study aims to address these two main questions. Additionally, the resident’s self-perceived proficiency levels for both procedures were assessed.

## Methods

European general surgery residents were invited to participate in this study from the 28th of July to the 20th of October 2019 and from the 1st of May to 1st of June 2020 by distributing the survey amongst members of the European Hernia Society and the Dutch Association of Surgical Residents. Participation was voluntary, and data was collected anonymously.

A 9-item, English-language web-based survey was developed to investigate the most commonly used learning methods, the perceived as most important learning methods and the resident’s self-perceived proficiency levels (Supplementary Material). The most commonly used learning methods were inquired by asking residents to select one or more methods that they had used to learn the IHR during their residency. For the importance of the learning methods a 5-point Likert scale was used to rate each learning method (1 = not at all important to 5 = very important).

**Fig. 1:**
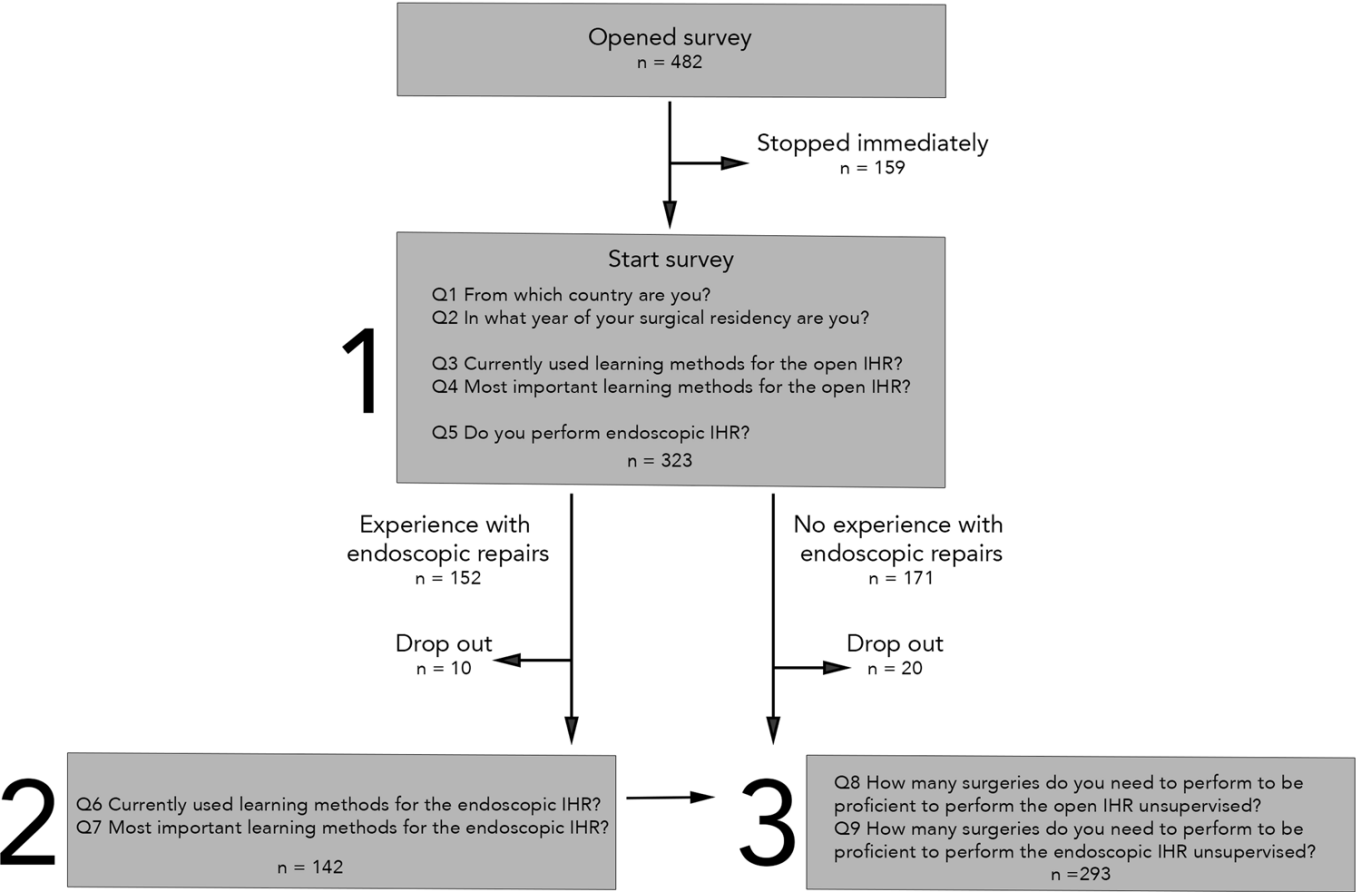
Study design

As shown in Fig. 1, the survey was split into three sections. The first section included questions regarding demographics (2 questions), currently used learning methods and what trainees perceived as the most important learning methods for the open IHR (2 questions) and whether the participant had experience with endoscopic IHR (1 question). The second section was exclusively for participants with endoscopic IHR experience and contained questions regarding currently used learning methods and what trainees perceived as the most important learning methods for the endoscopic repairs (2 questions). All participants were included in the third section, with questions concerning the supposed number of surgical procedures needed to achieve proficiency for the open and endoscopic IHR (2 questions).

Descriptive data of the currently used learning methods were presented using percentages. The descriptive data of the perceived importance of learning methods were presented as medians and interquartile range (IQR). Means were used for ranking these learning methods. All analyses were performed using SPSS® version 24.0 (IBM, Armonk, New York, USA).

## Results

In total, 482 general surgery residents opened the online survey, of whom 35 dropped out immediately and 323 completed the first section (Fig. 1). Hundred twenty-four residents completed the second section concerning endoscopic repair. Finally, 293 completed the proficiency questions in the third section. The surgical residents were on average in their third year of residency [2.0–5.0] and originated from 19 different countries, most of them were from Italy, the Netherlands and Spain (Table 1).

**Table 1:** Demographics

Participants	*n*	%
Total opened survey	482	
Open IHR section completed (1st)	323	67
Endoscopic IHR section completed (2nd)	124	26
Proficiency section completed (3rd)	293	61

From which country are you?	*n* = *323*	%
Italy	90	27.9
The Netherlands	66	20.4
Spain	65	20.1
United Kingdom	39	12.1
Denmark	19	5.9
Sweden	17	5.3
Czech Republic	7	2.2
Portugal	5	1.5
Germany	3	0.9
Greece	2	0.6
Austria	2	0.6
Macedonia	1	0.3
Romania	1	0.3
Poland	1	0.3
Ukraine	1	0.3
Ireland	1	0.3
Iceland	1	0.3
Albania	1	0.3
Estonia	1	0.3

Year surgical residency	*n* = *323*	%
1	53	16.4
2	51	15.8
3	65	20.1
4	49	15.2
5	60	18.6
6	45	13.9

Experience endoscopic IHR	*n* = *323*	%
No	171	52.9
Yes, supervised	77	23.8
Yes, unsupervised	75	23.2

**Fig. 2:**
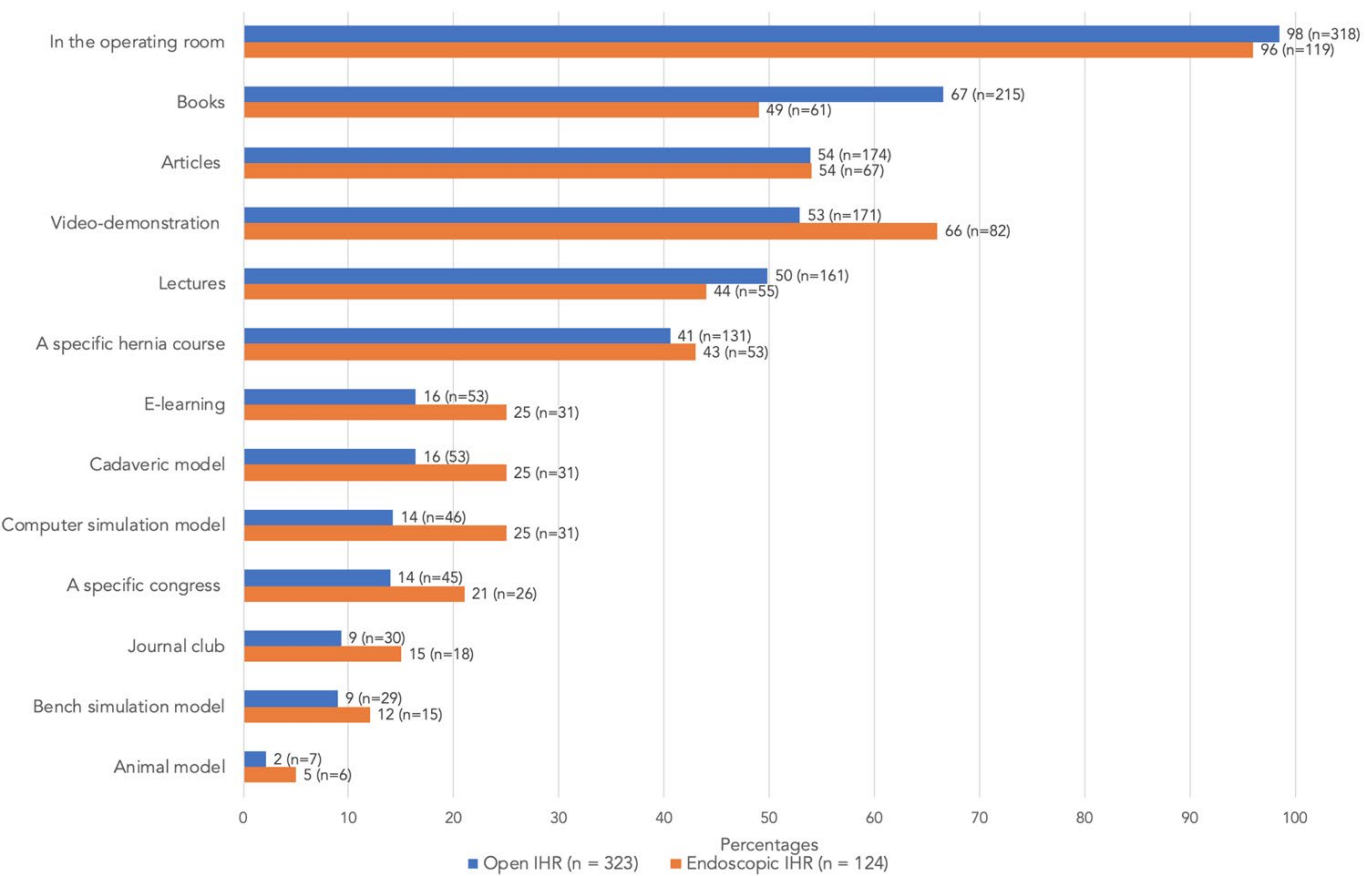
Currently used learning methods for the open and endoscopic inguinal hernia repairs

The five most commonly used learning methods for the open and endoscopic IHR were participation in the OR (98% and 96%, respectively), textbooks (67% and 49%, respectively), lectures (50% and 44%, respectively), video-demonstrations (53% and 66%, respectively) and journal articles (54% and 54%, respectively) (Fig. 2). The least used learning methods for the open and the endoscopic IHR were the use of the animal models (2% and 5%, respectively) and bench simulation model (9% and 12%, respectively).

As demonstrated in Table 2, what trainees perceived as the top three most important learning methods for the open and endoscopic IHR were; participation in the OR [5.00 (5.00–5.00) and 5.00 (5.00–5.00), respectively)], video-demonstrations [4.00 (4.00–5.00) and 4.00 (4.00–5.00), respectively], and hands-on hernia courses [4.00 (4.00–5.00) and 4.00 (4.00–5.00), respectively]. The two lowest-ranked learning methods for the open and endoscopic IHR were participation in a journal club [3.00 (2.00–4.00) and 3.00 (2.00–4.00), respectively] and practising on animal models [3.00 (2.00–4.00) and 3.00 (1.00–4.00), respectively].

**Table 2:** Open and endoscopic inguinal hernia repairs—importance of learning methods

	Open IHR (*n* = 323)			Endoscopic IHR (*n* = 124)		
	Median [IQR]	Mean	Rank	Median [IQR]	Mean	Rank
In the operating room	5.00 [5.00–5.00]	4.90	1	5.00 [5.00–5.00]	4.96	1
Video-demonstration	4.00 [4.00–5.00]	4.26	2	5.00 [4.00–5.00]	4.50	2
A specific hernia course	4.00 [4.00–5.00]	4.26	3	5.00 [4.00–5.00]	4.35	3
Cadaveric model	4.00 [3.00–5.00]	4.00	4	4.00 [3.00–5.00]	3.84	6
Articles	4.00 [4.00–4.00]	3.85	5	4.00 [3.00–5.00]	3.88	5
Lectures	4.00 [3.00–4.00]	3.83	6	4.00 [3.00–5.00]	4.06	4
Books	4.00 [3.00–4.00]	3.81	7	4.00 [3.00–5.00]	3.77	7
Bench simulation model	4.00 [3.00–5.00]	3.79	8	4.00 [3.00–5.00]	3.52	11
Computer simulation model	4.00 [3.00–4.00]	3.65	9	4.00 [3.00–5.00]	3.75	8
E-learning	4.00 [3.00–4.00]	3.58	10	4.00 [3.00–5.00]	3.67	9
A specific congress	3.00 [3.00–4.00]	3.50	11	4.00 [3.00–5.00]	3.64	10
Journal club	3.00 [2.00–4.00]	3.03	12	3.00 [2.00–4.00]	2.94	12
Animal model	3.00 [2.00–4.00]	2.99	13	3.00 [1.00–4.00]	2.78	13

**Fig. 3:**
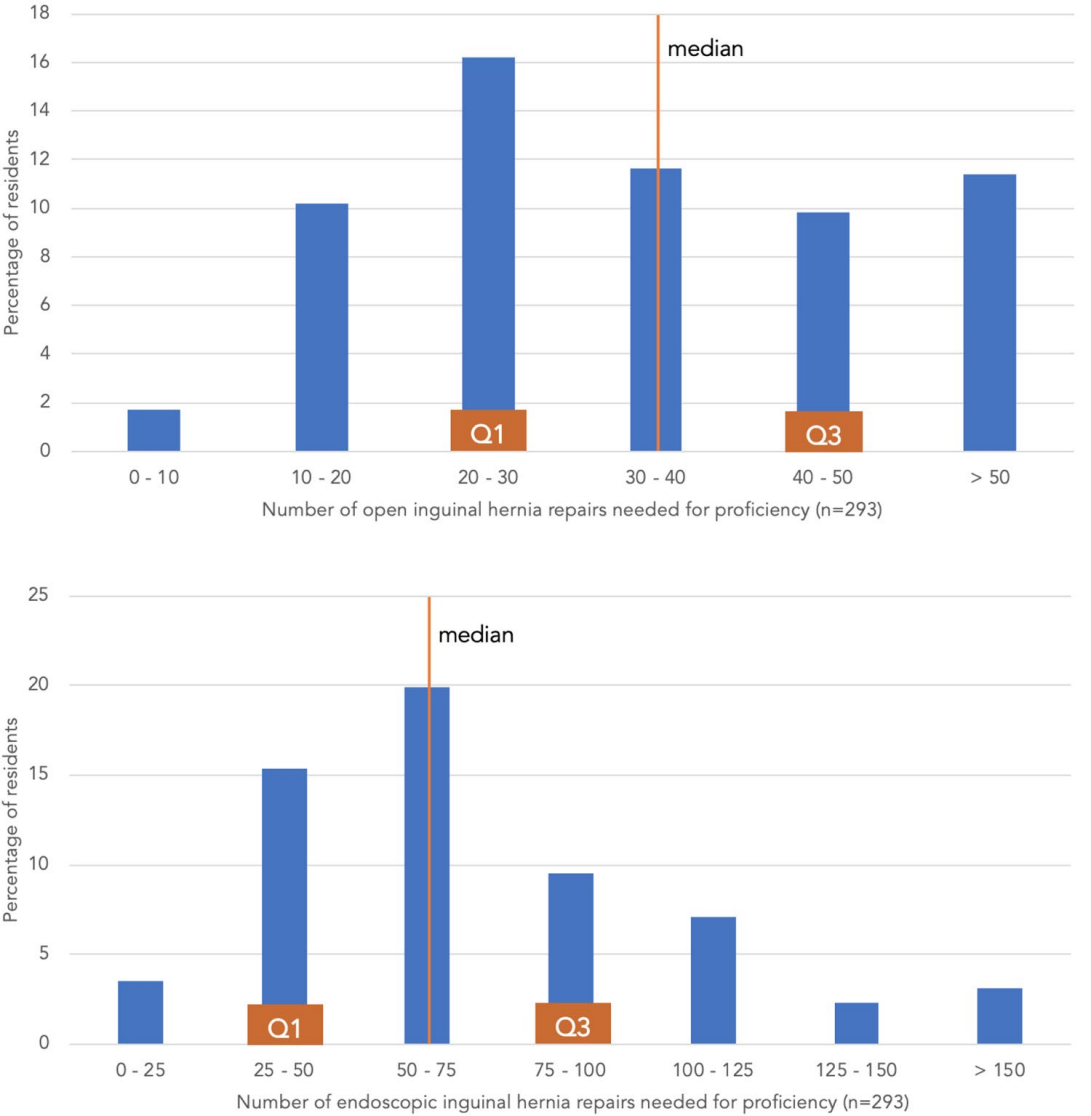
Estimated number of surgical procedures needed for proficiency in **a** open and **b** endoscopic inguinal hernia repair

The number of open IHR needed for proficiency was estimated by the surgical residents to be median 30 to 40 surgical procedures (range 20 – 50) (Fig. 3). The supposed proficiency number for the endoscopic IHR was median 50–75 surgical procedures (range 25–100).

## Discussion

The most frequently used learning methods in inguinal hernia surgery by surgical residents were participation in the OR, video-demonstrations, lectures, textbooks and articles, while the perceived most important learning methods were also participation in the OR, video-demonstrations and hands-on hernia courses. The residents prefer, besides the traditional learning methods, hands-on practice during specific hernia courses.

Currently traditional learning methods, video-demonstrations and learning in the OR are mainly used. Ideally, a resident is trained by observing the surgical procedure, then practising it in a safe environment and finally executing it in the OR while receiving feedback to improve further. A safe environment to practise their surgical knowledge and skills without compromising patient safety is provided by simulation training [13]. Several studies have proven the positive effects of simulation training [13–15], however, its implementation into the residents’ curriculum remains challenging [13]. The challenging implementation is underlined by our findings as the simulation methods are not frequently used (Fig. 2). Often, simulation training is unstructured or provided as ‘one-time’ events at courses [13]. The unstructured delivering of simulation training leads to not fully exploiting its potential, which would be the case in the aforementioned sequence practising in a simulation environment before operating on a patient. Despite the advantages of simulation, our participating surgical residents found the bench simulation model and computer simulation model to have low importance as learning methods for the IHR. The low importance is in contrast to a previous study in which 82% of surgical residents found simulation to be an important educational method for the IHR [6]. Although numerous bench simulation models [15, 16] and computer simulation models have been validated for the IHR [17–19], we wonder if the unfamiliarity of the participating surgical residents with these bench simulation model or computer simulation model could explain the perceived low importance of these learning methods. These validated simulation models should find their way to day-to-day use for IHR training.

Video-demonstrations were mentioned as one of the most important learning methods in our study. Zahiri and colleagues found video-demonstrations to be important for 87% of surgical trainees [6]. An advantage of online videos is that they can be accessed on demand by surgical residents—any time and any place—known as the just-in-time principle [20]. YouTube is the most preferred streaming video source among medical students, surgical residents and faculty members [21]. However, in general and especially if the contributor is unknown, surgical videos on YouTube lack educational value and may display inadequate or even unsafe manoeuvres [22–24]. As YouTube is not a peer-reviewed platform, videos are ranked on popularity and not on quality [25]. WebSurg is another online platform for open source videos of minimally invasive surgical procedures only [26]. The WebSurg videos regarding the total extraperitoneal procedure for IHR were found to be of suboptimal quality in terms of educational value [27]. Teaching grade video-demonstrations of surgical procedures should be peer-reviewed and have high educational value [22, 24, 27]. A rather new online surgical educational platform is Incision Academy with surgical videos containing standardized procedural steps [28] and of which the content has been supervised by surgeons and anatomists.

Surgical training is aimed at reaching a proficiency level in performing a surgical procedure independently. In this study, surgical residents were asked to indicate how many procedures they need to become proficient in the IHR. Our participating surgical residents estimated 30–40 procedures (range 20–50) were required to achieve proficiency in open IHR. In previous studies, around 40 open IHR [1], or even 64 repairs were needed for proficiency [29]. In our survey, the estimated number of endoscopic IHR needed to become proficient were 50–75 surgical procedures (range 25–100). Previous study indicated that more than 100 endoscopic repairs are required to achieve outcomes comparable to open anterior mesh repair [5]. However, in-line with our results, other articles referred to 65 procedures as a minimum volume necessary to train for endoscopic inguinal hernia repairs [30, 31]. Due to this discrepancy between the numbers estimated for proficiency by our surgical residents and the numbers needed for proficiency, the question arises whether surgical residents overestimate themselves, or the trainees underestimate the residents. Some surgical residents require less surgical procedures than others to achieve proficiency [32]. A comprehensive yet easy to use assessment tool should be used to assess the performance of a surgical procedure, and to indicate one's proficiency more accurate. Possible options could be competence tracking using Observational Clinical Human Reliability Assessment (OCHRA) or Surgical Quality Assurance (SQA) [33, 34].

### Future perspectives

The sequence of a surgical residents’ training—observing, practising, performing and reflecting on a surgical procedure—should be facilitated. First to facilitate observing of surgical procedures, accurate video-demonstrations should be provided. Secondly, as the learning yield of surgical simulation training is promising, the perceived low importance amongst surgical residents should be explored. Perhaps the familiarity of qualitative simulation models is lacking to incorporate simulation training into surgical residents' training programs. Especially, the timing of the simulation trainings should be optimized so a resident can train in a safe environment and then progress to performing the surgical procedure in the OR. Finally, to facilitate the reflection on a surgical procedure and to evaluate the residents’ proficiency, the applicability of the OCHRA or SQA should be further researched.

### Limitations

This study has a number of limitations that need to be considered. Of 482 surgical residents that opened the survey, 159 residents stopped immediately. It is possible that these surgical residents had different views on learning methods. Second, the majority of the residents originated from Italy, the Netherlands and the Spain (*n* = 221 of 323) which might have made the results less representative for Europe, although the participants from the various European countries indicated similar experienced and preferred learning modalities. Third, to keep our survey short and concise, we surveyed the learning methods without specifying which learning goal was desired, such as theoretical knowledge or technical skills. We also did not ask how many open or endoscopic surgical procedures the residents had performed.

## Conclusion

In conclusion, this study demonstrated a discrepancy between learning methods that are currently used by surgical residents to learn the open and endoscopic IHR and preferred learning methods by them. There is a need for more emphasis on practising before entering the OR. To achieve this more simulation models for IHR are needed. This would support surgical residents’ training by first observing, then practising and finally performing the surgery in the OR. It is highly recommended to implement simulation based training in educational residency programs.

## Electronic supplementary material

Below is the link to the electronic supplementary material.

Supplementary file1 (PDF 51 kb)
