# Supplementary material for: Learning inguinal hernia repair? A survey of current practice and of preferred methods of surgical residents
Source: Hernia. 2020 Sep 5;24(5):995–1002. doi: 10.1007/s10029-020-02270-y (PMC7520418; doi:10.1007/s10029-020-02270-y)
Supplement: Supplementary file 1 — Supplementary file1 (PDF 51 kb) [file 10029_2020_2270_MOESM1_ESM.pdf]

Supplementary Material - Survey

Q1. From which country are you?

Q2. In what year of your surgical residency are you?

- 1
- 2
- 3
- 4
- 5
- 6

Q3. Which type of educational methods have you experienced to train for the open inguinal hernia repair? (multiple options are possible)

- Journal club
- Lectures
- Computer simulation model
- Bench simulation model made from fabric, 3D printing or other synthetic material (skills lab simulation models)
- Cadaveric model
- Animal model
- In the operating room
- A specific hernia course
- A specific congress
- Video-demonstration of the operation
- E-learning
- Books

Q4. How important do you find the following educational methods to train for the open inguinal hernia repair? (5-point Likert scale; 1 = not important, 3 = neutral, 5 = very important)

- Journal club
- Lectures
- Computer simulation model
- Bench simulation model made from fabric, 3D printing or other synthetic material (skills lab simulation models)
- Cadaveric model
- Animal model
- In the operating room
- A specific hernia course
- A specific congress
- Video-demonstration of the operation
- E-learning
- Books

Q5. Do you perform the endoscopic inguinal hernia repair?

- Yes, unsupervised
- Yes, supervised
- No

Q6. Which type of educational methods have you experienced to train for the endoscopic inguinal hernia repair? (multiple options are possible)

- Journal club
- Lectures
- Computer simulation model

- Bench simulation model made from fabric, 3D printing or other synthetic material (skills lab simulation models)
- Cadaveric model
- Animal model
- In the operating room
- A specific hernia course
- A specific congress
- Video-demonstration of the operation
- E-learning
- Books

Q7. How important do you find the following educational methods to train for the endoscopic inguinal hernia repair? (5 point Likert scale; 1 = not important, 3 = neutral, 5 = very important)

- Journal club
- Lectures
- Computer simulation model
- Bench simulation model made from fabric, 3D printing or other synthetic material (skills lab simulation models)
- Cadaveric model
- Animal model
- In the operating room
- A specific hernia course
- A specific congress
- Video-demonstration of the operation
- E-learning
- Books

Q8. How many surgeries do you think you need to perform to be proficient to perform the open inguinal hernia repair unsupervised?

- 0 – 10
- 10 – 20
- 20 – 30
- 30 – 40
- 40 – 50
- > 50

Q9. How many surgeries do you think you need to perform to be proficient to perform the endoscopic inguinal hernia repair unsupervised?

- 0 – 25
- 25 – 50
- 50 – 75
- 75 – 100
- 100 – 125
- 125 – 150
- > 150
